# UV-Light Exposed Prion Protein Fails to Form Amyloid Fibrils

**DOI:** 10.1371/journal.pone.0002688

**Published:** 2008-07-16

**Authors:** Abhay Kumar Thakur, Ch Mohan Rao

**Affiliations:** Centre for Cellular and Molecular Biology, Council of Scientific and Industrial Research, Hyderabad, Hyderabad, India; Massachusetts Institute of Technology, United States of America

## Abstract

Amyloid fibril formation involves three steps; structural perturbation, nucleation and elongation. We have investigated amyloidogenesis using prion protein as a model system and UV-light as a structural perturbant. We find that UV-exposed prion protein fails to form amyloid fibrils. Interestingly, if provided with pre-formed fibrils as seeds, UV-exposed prion protein formed amyloid fibrils albeit with slightly different morphology. Atomic force microscopy and electron microscopic studies clearly show the formation of fibrils under these conditions. Circular dichroism study shows loss in helicity in UV-exposed protein. UV-exposed prion protein fails to form amyloid fibrils. However, it remains competent for fibril extension, suggesting that UV-exposure results in loss of nucleating capability. This work opens up possibility of segregating nucleation and elongation step of amyloidogenesis, facilitating screening of new drug candidates for specifically inhibiting either of these processes. In addition, the work also highlights the importance of light-induced structural and functional alterations which are important in protein based therapeutics.

## Introduction

Transmissible Spongiform Encephalopathy is a group of diseases such as Kuru, Creutzfeldt - Jakob disease (CJD), Gerstmann-Straussler Syndrome (GSS), and Fatal Familial Insomnia (FFI) characterized by neurodegeneration and deposition of amyloid plaques. Conformational transition of cellular prion protein (PrP^c^) is believed to be the major cause for these diseases [1]. It took several decades of research for the evolution of concepts from the slow virus hypothesis [2] to the prion protein hypothesis [1], [3]. However, despite extensive research, the controversy regarding the source of infectious agent is not yet resolved. The role of nucleic acids [4], [5], polyanions [6], [7] and lipids [8]–[10] is being investigated, in addition to the transformed prion protein PrP^Sc^ in the pathogenesis.

Conformational conversion of alpha-prion protein (PrP^c^) to beta-prion protein (PrP^Sc^), accompanied by aggregation, leads to amyloid fibril formation. Mechanistically, amyloidogenesis or the process of amyloid formation involves three major stages - structural perturbation, nucleation and fibril extension. Nucleation process is the rate-limiting step due to kinetically disfavored oligomerization (self assembly) of intermediates. These assemblies are partially concentration-dependent [11] and show presence of hydrophobic cooperativity in the process [12]. This rate-limiting phase is reflected as the lag phase in kinetics of amyloid formation. The lag in kinetics persists till the formation of a critical nucleus, after which the reaction proceeds in favor of a rapid increase in size [13]. Bidirectional growth of elongating fiber was observed in this stage [14]. Binding of monomer to continuously growing fiber and subsequent conformational change characterize this event [15], [16]. These amyloid aggregates show congo-red birefringence and cross-β-sheet structure. The organization of these fibrils remains the same amongst different types of proteins- unbranched 2–3 subprotofibrils (10–15 Å) helically arrange to form protofilaments (protofibril) (25–30 Å), which associate laterally or twisted in bundle of five to form mature fibrils [17].

The pre-nucleation stage involves structural perturbation and destabilization of the native state, thus forming non-native states or partially unfolded intermediates (kinetic or thermodynamic intermediates), which are prone to aggregation. Mild to harsh conditions such as low pH [18], exposure to elevated temperatures [19], [20] exposure to hydrophobic surfaces [21], partial denaturation using urea [19], [22] and guanidinium chloride [23], are used to achieve non-native states. However, natively unfolded proteins such as α-synuclein, tau protein, yeast prion, require structural stabilization for the formation of partially folded intermediates, which are competent for fibril formation. Conditions for partial stabilization include low pH [24], presence of SDS [25], elevated temperature or chemical chaperones [26].

We have tested the possibility of UV exposure as a structural perturbant to initiate nucleation leading to amyloid fibril formation or aggregation using the mouse full-length prion protein (PrP 23-231) as a model system. We find that UV exposure of PrP leads to structural changes and amorphous aggregation. UV-exposed PrP fails to form amyloid fibrils. Interestingly, however, it remains competent for fibril extension if provided with pre-formed fibrils as seeds. UV exposure, thus, appears to provide a novel handle to segregate nucleation and fibril extension.

## Results

### Photo-aggregation of prion protein

**Figure 1:**
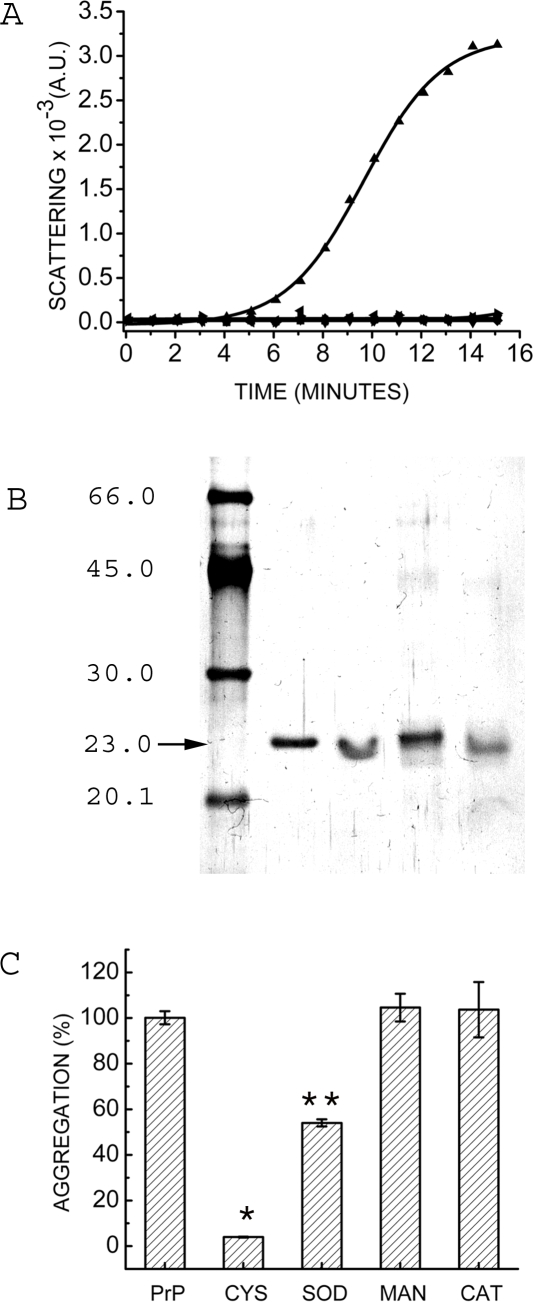
Photo-aggregation of prion protein. A) Mouse full-length prion protein (2.6 µM) in 50 mM phosphate buffer was exposed to 290 nm of light. Scattering was measured by setting excitation and emission monochromators at 465 nm (see materials and methods). Photo-aggregation of prion protein at 290 nm (▴). Prion protein was exposed to light of various wavelengths in above conditions such as 214 nm (▪), 350 nm (▾) and 400 nm (♦). Prion protein was exposed to light of 290 nm under amyloid condition (3 M urea and 1 M GdmCl) (◂) and unexposed prion protein under amyloid condition (▸). B) SDS PAGE of prion protein Lane1- Low Molecular weight marker (GE healthcare, UK); Lane 2-Purified recombinant mouse full-length prion protein with β-mercaptoethanol; Lane 3- Purified recombinant mouse full-length prion protein without β-mercaptoethanol; Lane 4- Photo-aggregated prion protein with β-mercaptoethanol; Lane 5- Photo-aggregated prion protein without β-mercaptoethanol. Bands were visualized by silver staining. C) Extent of aggregation in the presence of antioxidants is represented as bars. Percent aggregation was calculated with respect to aggregation of prion protein alone. All experiments were done at room temperature. Prion protein (PrP) was used at a concentration of 2.6 µM. The concentrations of the antioxidants used for inhibition of aggregation were of (CYS) L-cysteine, 1 mM; (SOD) Superoxide Dismutase, 20 µg/ml (∼64 U/ml); (MAN) Mannitol, 50 mM and (CAT) Catalase, 2.5 ng/ml (∼0.895 mU/ml). * represents p<0.005: ** represents p<0.01 (using student paired‘t’ test).

Mouse full-length prion protein was exposed to near-UV light of 290 nm under constant stirring. Figure 1A (triangles) shows aggregation profile of prion protein upon exposure to UV light. The curve shows lag phase of 4–5 minutes. The lag phase of this aggregation indicates its nucleation-dependent behavior. Interestingly, upon incubation with Thioflavin T, a specific amyloid binding fluorescent probe, we did not observe enhanced fluorescence, suggesting the formation of unordered or amorphous aggregates (data not shown). It was further confirmed with TEM where we did not observe the presence of any fibrils (data not shown). We have exposed the protein to UV-light of 214, 350 and 400 nm and found no aggregation (Figure 1A).

In order to investigate the nature of this aggregation, we have carried out aggregation in presence of urea and SDS. Upon addition of 0.1% SDS in the completely aggregated sample, we observed loss of scattering within a short period of time, indicating non-covalent nature of this aggregates. Amyloidogenesis of prion protein can be induced in 3 M urea and 1 M GdmCl with prolonged incubation and /or addition of pre-formed fibrils as seeds. Prion protein, in 3 M urea and 1 M GdmCl did not show any aggregation during the period of experiment (Figure 1A). Exposing the protein to UV-light of 290 nm, under these conditions, also did not lead to increase in Rayleigh scattering (Figure 1A) further, corroborating non-covalent nature of aggregation. Intramolecular disulphide bond is shown to have a role in the amyloidogenic process in β2-microglobulin and prion protein [27], [28]. Our preparation of full length mouse prion protein (23-231) has an intact intramolecular disulphide bond (as checked by the SDS-PAGE with and without β-mercaptoethanol). We have checked the disulphide bond after the sample is exposed to UV-light. SDS-PAGE of photo-aggregated prion protein, in the presence and absence of β-mercaptoethanol is identical indicating no alteration of the existing disulphide bond (Figure 1B).

### Prevention of photo-aggregation by antioxidants

Upon near UV exposure, tryptophan undergoes photo oxidation and generates N-formylkynurenine, kynurenine, and tryptamine as oxidative products. Oxidation of protein in aerated aqueous solvent leads to generation of several reactive oxygen species and causes aggregation of protein. In order to understand the molecular mechanism of the photo-aggregation of prion protein, we have monitored this aggregation in the presence of several antioxidants. Inhibition of amorphous aggregation in the presence of antioxidants indicates quenching/scavenging of corresponding radicals thus, showing its involvement in this process. We used several antioxidants specific for different oxygen species such as L-cysteine for singlet oxygen, superoxide dismutase (SOD) for superoxide, mannitol for hydroxyl radical and catalase for peroxyl radical.

Figure 1C shows prevention of amorphous aggregation by antioxidants. We observed maximum aggregation in the absence of antioxidants. We see no effect on addition of 50 mM mannitol; aggregation profiles in the absence and the presence of mannitol superimpose within the experimental error, suggesting that hydroxyl radicals perhaps are not involved in the photo-aggregation of prion protein. Even catalase had no effect on the extent of aggregation (Figure 1C) suggesting that peroxyl radicals do not appear to have any role in the photo-aggregation of prion protein. Superoxide dismutase however had some effect on the aggregation process. Upon addition of 160 U of superoxide dismutase (SOD), we observed ca. 45% of inhibition (∼55% of aggregation) compared to the aggregation of prion protein in the absence of any antioxidants (Figure 1C). This inhibition suggests some role for the superoxide ions in the photo-aggregation process. Interestingly, presence of 1 mM of free amino acid L-cysteine was able to completely abrogate this aggregation (97% inhibition) (Figure 1C) suggesting the major role for singlet oxygen in the photo-aggregation process. Taken together, our results show that peroxyl and hydroxyl radicals do not have any role in the photo-aggregation of prion protein. Superoxide has significant (45%) and singlet oxygen has dramatic (97%) effect on the photo-aggregation of prion protein.

### UV-exposure alters conformation of prion protein

**Figure 2:**
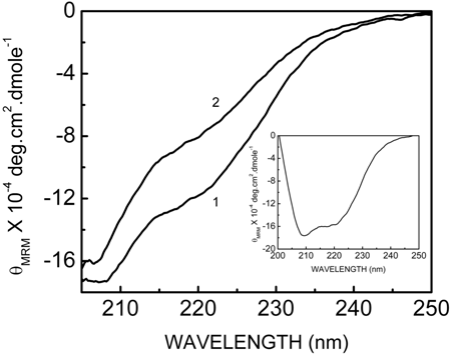
Secondary structural changes of prion protein upon UV-exposure. Far UV CD spectra of prion protein (43.4 µM) in amyloid forming condition (3 M urea, 1 M GdmCl, 100 mM NaCl, 20 mM phosphate buffer, pH 6.8) before (curve 1) and after (curve 2) exposure to UV light. Inset: Far UV CD spectrum of prion protein (43.4 µM) in phosphate buffer.

Prion protein shows predominance of alpha helices by far UV CD measurements (Figure 2 inset). Due to aggregation of prion protein upon UV-light exposure CD measurements were not possible. However, we could record CD spectra of the UV-exposed prion protein in amyloidogenic conditions (3 M urea and 1 M GdmCl). Under these conditions, exposure to UV-light leads to some loss of helical structure (Figure 2 curve 1). The photo-aggregation process of prion protein shows a lag period of 4–5 minutes (Figure 1A) indicating accumulation of aggregation-prone intermediates. Thus, we UV-exposed prion protein for five minutes under partial denaturing, amyloidogenic conditions. CD spectrum was recorded soon after the exposure. UV exposure of about five minutes was sufficient to cause observable differences in the far UV CD. The CD spectrum in Figure 2 (curve 2) shows significant decrease in the helicity upon UV exposure in comparison to prion protein under amyloidogenic condition. Due to enhanced light scattering by urea and guanidinium chloride, we restricted CD measurements to above 205 nm. Thus, the CD spectral study clearly indicated the structure perturbing effect of brief exposure to UV light.

### UV-exposed prion protein fails to form amyloid *de novo*

**Figure 3:**
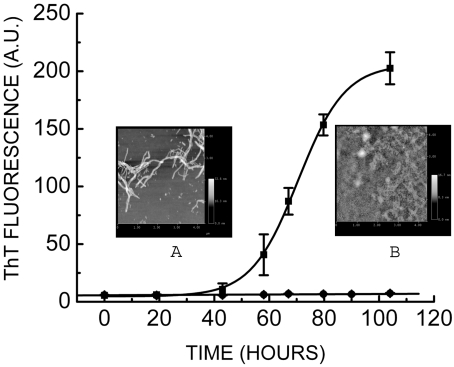
Effect of UV-exposure on amyloid fibril formation of prion protein. Amyloid fibril formation of prion protein (43.4 µM) (▪) and UV-exposed prion protein (43.4 µM) (•) monitored by increase in ThT fluorescence (see materials and methods). Inset: AFM image of A) prion protein amyloid fibrils (43.4 µM) and B) UV-exposed prion protein samples (43.4 µM).

In order to study the consequence of such structural perturbation (accumulation of non-native states) on the amyloidogenic process, we investigated amyloidogenesis of UV-exposed prion protein. Enhanced ThT fluorescence can be measured to monitor the process of amyloid fibril formation. Figure 3 (filled squares) shows ThT fluorescence as a function of time of prion protein (43.4 µM) in the presence of 3 M urea, 1 M GdmCl and 150 mM NaCl at pH 6.8 at 37°C, (∼1 mg/ml), under constant shaking at 600 rpm. As can be seen from the Figure 3 (filled squares), the lag phase extends to 48 hrs after which ThT fluorescence increases and attains saturation within 120 hours. Thus, mouse full-length unexposed prion protein forms amyloid fibrils *de novo* in above conditions. Surprisingly, incubation of UV-exposed prion protein under identical amyloid forming conditions did not result in any increase in ThT fluorescence (Figure 3 filled circles). Even after incubation for several days, UV-exposed prion protein did not show fibrils in it. We checked this sample under AFM to further confirm absence of any fibrils in this sample. Figure 3 inset A shows amyloid fibrils of unexposed prion protein formed *de novo* as observed in scanning AFM. AFM of UV-exposed prion protein does not show formation of fibrils (Figure 3 inset B). Thus, both ThT fluorescence and AFM clearly demonstrate the inability of UV exposed prion protein to form amyloid fibrils *de novo*.

**Figure 4:**
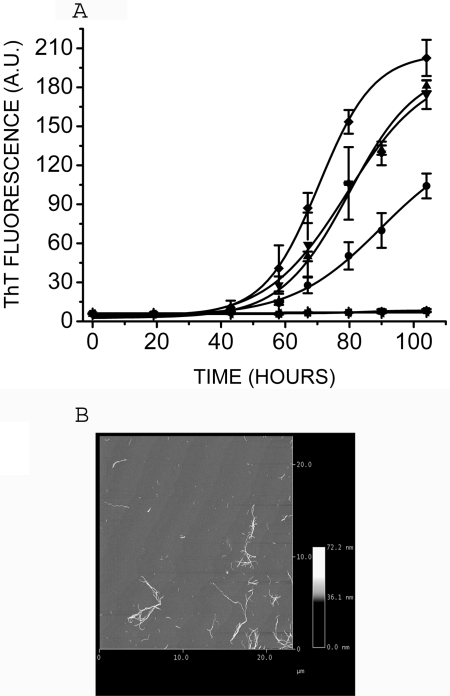
A) Concentration dependence of prion protein for *de novo* amyloid formation. Different concentrations of unexposed prion protein 4.34 µM (▪), 10.85 µM (•), 21.7 µM (▴), 32.5 µM (▾), 43.4 µM (♦) and 43.4 µM (◂) of UV-exposed prion protein were subjected to amyloid forming conditions. (n = 3) B) AFM image of amyloid fibrils of prion protein (10.85 µM) incubated in amyloid forming buffer. Fibrils are formed even at this low concentration.

The inability of UV-exposed prion protein to form amyloid fibrils is intriguing. In order to see if UV exposure causes loss of available protein leading to sub-critical level, if any, we have investigated the concentration dependence of prion protein in its amyloidogenesis. Several concentrations ranging from 4.34 µM (∼0.1 mg/ml) to 43.4 µM (∼1 mg/ml) of unexposed prion protein were prepared for amyloid formation. All the samples were subjected to amyloid forming conditions as described in materials and methods. We observed rise in ThT fluorescence after 48 hrs in 43.4 µM unexposed prion protein sample (Figure 4A). Even at one fourth of the initial concentration (*i.e.*, even if the loss were upto 75%) samples show significant increase in ThT fluorescence indicating amyloid fibril formation. Fibrils could be seen at this concentration in AFM as shown in Figure 4B. Fibril formation could be seen at dilution as low as ten-fold (4.34 µM) (data not shown). However, UV-exposed prion protein at a very high concentration (43.4 µM) showed no fibril formation as monitored by ThT fluorescence and AFM. Increase in ThT fluorescence and the presence of fibrils in the samples (4.34 µM, 10.85 µM); indicate that one fourth or even one tenth of the concentration used for the experiment is sufficient for amyloid formation, ruling out the trivial possibility of loss of protein as a possible cause for the observed lack of amyloid formation with the UV-exposed samples.

### UV-exposed prion protein forms amyloid upon seeding

**Figure 5:**
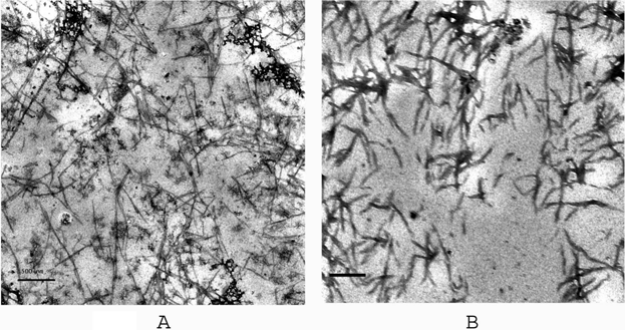
EM images of fibrils of seeded reactions. Small amount of sample was placed on copper grid and stained by uranyl acetate for EM imaging (see materials and methods). EM image of A) prion protein and B) UV-exposed prion protein. Scale bar – 500 nm.

**Figure 6:**
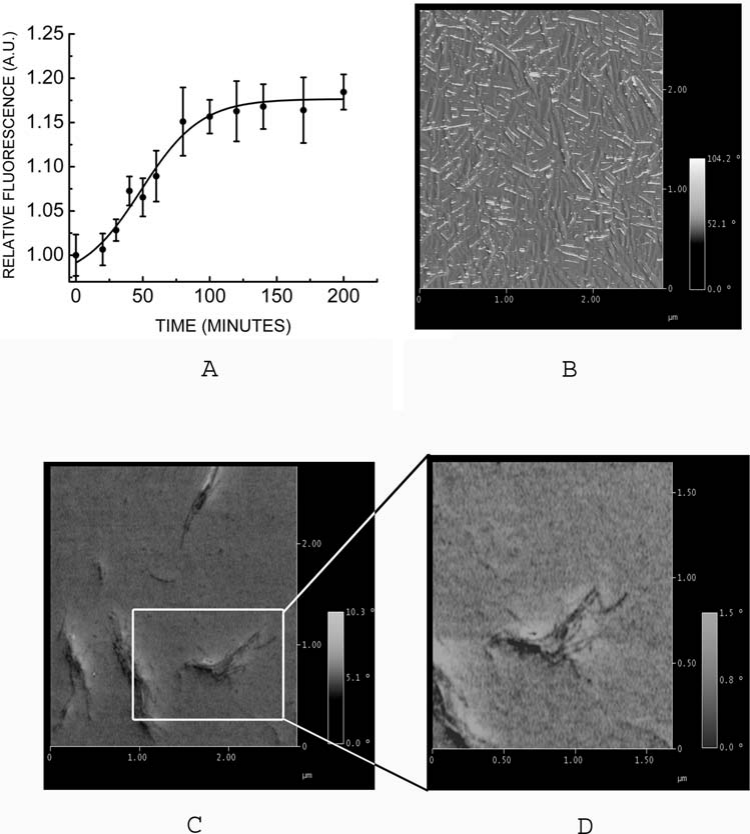
ThT fluorescecence and phase images of fibrils of seeded reactions. A) ThT fluorescence of seeded reaction of UV-exposed prion protein using seeds of unexposed prion protein. Phase image of B) protein after seeding reaction and C) UV-exposed prion protein after seeding. D) Enlarged phase image of UV-exposed prion protein after seeding.

One of the hallmark features of amyloid formation is seeding reaction where fragments of amyloid fibrils of protein act as seed when mixed with monomer protein and leads to fibril extension. Seeded fibril extension reactions have no lag periods in contrast to *de novo* fibril formation. Seeding eliminates the need for nucleation. UV-exposed prion protein loses its ability to form amyloid fibrils. Does the UV-exposed protein remain competent for fibril extension, under conditions where seeding is not important? In order to test this possibility, we have generated fibrils from the prion protein and sonicated them to make seeds and used them with UV-exposed prion protein. Interestingly, UV-exposed protein, with seeding, indeed showed significant increase in ThT fluorescence (Figure 6A) indicating fibril formation. This is an interesting result as this protein failed to form fibrils *de novo* (as described earlier) but continued to elongate in the presence of seeds of amyloid fibrils obtained from unexposed prion protein. Figure 5A and B shows EM images of UV-exposed prion protein compared with that of unexposed prion protein amyloid fibrils formed in the presence of seeds.

### UV-exposed prion protein fibrils show altered fibril morphology

We further investigated fibrils morphology under these conditions using electron microscopy and atomic force microscopy. Fibrils formed from monomers of unexposed prion protein in the seeded reaction were slender and long as shown in Figure 5A. These fibrils showed canonical organization of fibrils with subprotofibrils of 8.89±0.355 nm twisting around each other to form protofilaments of 20.57±0.833 nm (determined as described in materials and methods). Contrary to this, fibrils obtained from monomers of UV-exposed protein in seeded reactions were thick and stout and flat in appearance and showed thickness of 30±0.916 nm and 47.72±2.066 nm indicating different organization of fibrils as observed from EM image (Figure 5B). We have recoded phase images in tapping mode AFM. Phase image was obtained to find compactness of molecules under imaging. Compactness (or stiffness) refers to hardness or softness of the sample. Hard samples give larger change in phase angle; soft samples in contrast lead to smaller changes in phase angle (please see materials and methods). The fibrils of unexposed prion protein show phase of 37.5±0.358° as observed from phase image (Figure 6B) (determined as described in experimental procedure). In contrast, phase images of fibrils of UV-exposed prion protein showed significantly low phase of 3.82±0.1457° (Figure 6C and D).

Significantly lower phase for UV-exposed prion protein fibrils indicates less compact packing (or less stiffness) of these fibrils. These results illustrate significant change in morphology and packing of fibrils upon UV-exposure.

## Discussion

Protein misfolding and aggregation is one of the major causes of several neurodegenerative diseases and myopathies. Molecular understanding of the ordered aggregation, amyloid fibril formation, is critical for designing strategies to mitigate the problem. Perturbing the system with denaturants such as urea and guanidinium hydrochloride, lower pH etc have provided wealth of information on amyloidogenic process. We have tested the possibility of using UV exposure for such structural perturbation that might initiate amyloid fibril formation. Interestingly, however, we find that UV-exposed prion protein fails to form amyloid fibrils *de novo*. We have demonstrated that this observed failure to form amyloid fibrils is not due to loss of protein (photochemical degradation) leading to sub-critical levels for fibril formation. We find that UV-exposure of prion protein leads to loss of helicity. Interestingly, UV-exposed prion protein forms fibrils when provided with nucleus (pre-formed fibril fragments as seeds). This clearly shows that UV-exposure leads to failure of nucleation, separating nucleation from fibril extension.

We have exposed prion protein to UV light of 290 nm. At this wavelength, tryptophan is the major chromophore. Other aromatic residues and cysteine also absorb UV light however to a lesser extent. Generation of reactive oxygen species, conformational change and photo-aggregation of γ-crystallin, an eye lens protein, are well documented [29], [30]. In addition to photo-aggregation of γ-crystallin, we have earlier observed conformational dependence of the photo vulnerability of tryptophan residues in different proteins [31]. Excitation of tryptophan and tyrosine leads to generation of tryptophanyl and tyrosinyl radicals. Tryptophanyl radicals lead to formation of several tryptophan oxidative products such as N-formylkynurenine (NFK), Kynurenine and tryptamine. These products, especially NFK are efficient endogenous sensitizers and at near UV light generate singlet oxygen and superoxide radicals [32], [33]. Such photochemical processes could lead to photo-aggregation of prion protein as we observed. Prevention of aggregation by L-cysteine, a scavenger of singlet oxygen species [34]–[37] and SOD suggests that the main causative agents are singlet oxygen and superoxide.

Oxidation is one of the factors associated with the prion disease. Increased levels of oxidation (including glycoxidation, peroxidation, and protein nitration) [38]–[40], free radical-mediated DNA damage [41] and glial activation [42] have been observed in sporadic CJD cases. Moreover, oxidative stress was shown to exist in early stage of prion invasion indicating it as a causative early agent [43]. These evidences indicate that prion protein is present in redox-active environment and is actively involved in redox activity. This might lead to exposure of prion protein to various oxidative insults. A recent study showed highly localized chemical modification of prion protein upon oxidation mediated by hydroxyl radical [44]. Another effect of oxidation is shown as degradation/specific cleavage of prion protein [45], [46]. We find that upon UV-induced oxidation, prion protein leads to changes in secondary structure- decrease in alpha-helical content. Possibly, photo-oxidation of aromatic amino acids might lead to side chain modification leading to conformational change and thus prion protein becomes prone to amorphous aggregation.

Interestingly, we find that UV-exposed prion protein fails to form amyloid fibrils under the condition in which the unexposed prion protein readily forms amyloid fibrils. The possible reasons for the failure of exposed prion protein to form amyloid fibrils could be: 1) photochemical damage causing loss of available protein leading to sub-critical level, if any, of prion protein for amyloidogenesis, 2) incapability of UV-exposed prion protein to participate in amyloid process probably due to loss of crucial structure of monomers and 3) inhibitory effect of oxidized molecule on amyloid nucleus. Our concentration-dependent studies rule out the possibility of sub-critical protein concentration. We find that UV-exposed protein, if provided with pre-formed seeds, readily forms amyloid fibrils, thus ruling out the possible inhibitory effects of photo oxidized molecules. It appears that UV-exposure renders prion protein incapable of forming amyloid nucleus. This clearly shows that UV-exposure leads to failure of nucleation, separating nucleation from fibril extension.

**Figure 7:**
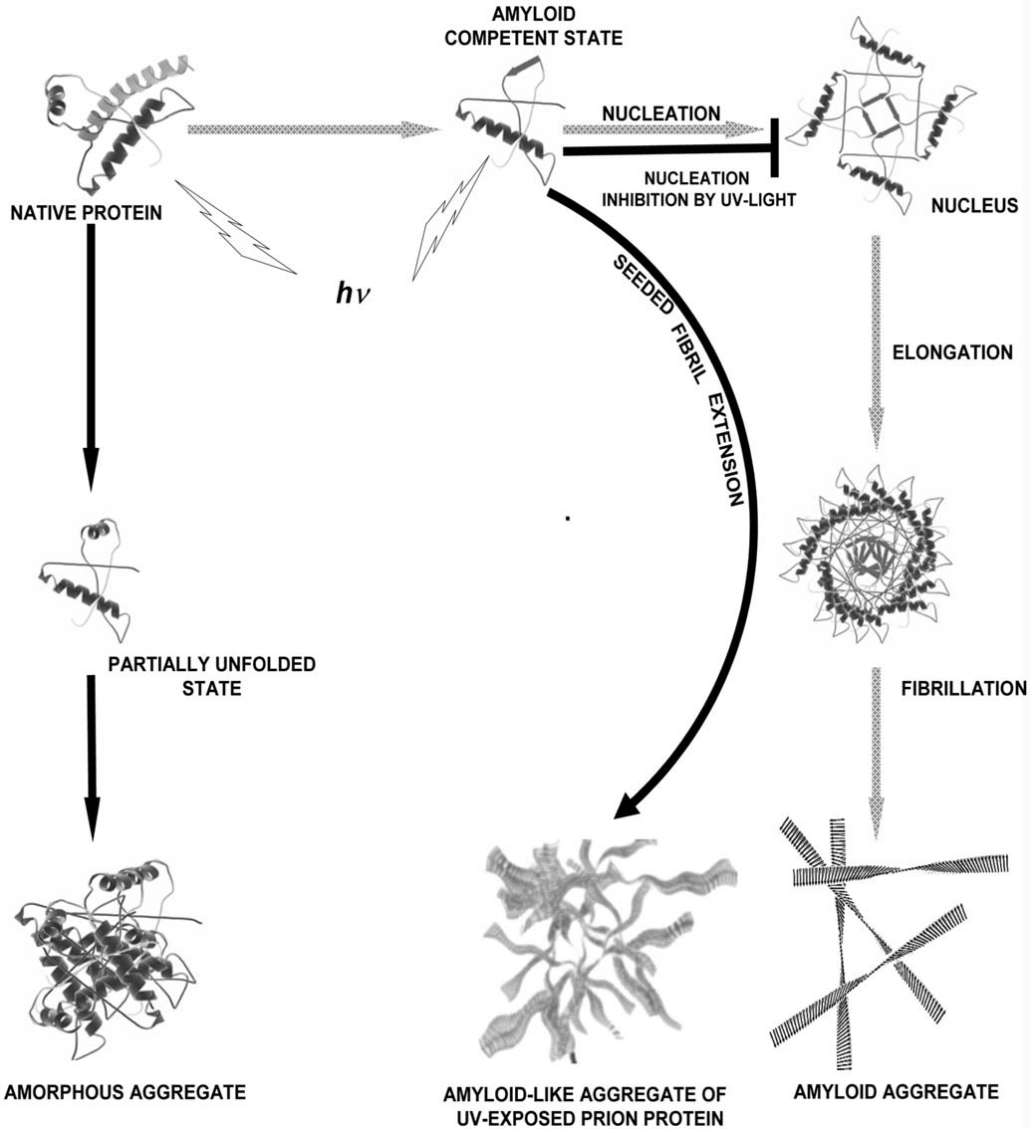
Schematic representation of effect of light on amyloid fibril formation of prion protein. (Please note the structures are schematic representations).

Figure 7 schematically describes the effect of light on the amyloidogenesis. Native, largely alpha helical, prion protein under amyloidogenic conditions undergoes structural changes and forms amyloid nucleus to which other monomers join to extend the nucleus to protofibrils and subsequently thicker fibrils and amyloid aggregates (grey arrows). UV-exposure inhibits the nucleation process hence fibril formation. UV-exposed prion protein undergoes some structural alterations and forms amorphous aggregates. However, it remains competent for fibril extension reaction if provided with pre-formed fibril seeds as nucleus albeit with slightly different morphology (Fig 7, dark arrows).

Misfolding and aggregation of proteins are believed to be the major cause of neurodegeneration disorders such as Alzheimer's, Parkinson and prion disease. Therapeutic approaches to intervene the process met with very little success [47]–[50]. Current therapeutic approaches do not address nucleation and elongation steps separately. Our work opens up possibility of segregating nucleation and elongation step of amyloidogenesis, facilitating screening of new drug candidates for specifically inhibiting either of these processes. In addition, the work also highlights the importance of light induced structural and functional alterations which are important in protein based therapeutics.

## Materials and Methods

All chemicals and reagents were of analytical or ultra pure grade. IPTG was obtained from Bangalore Genei (India), Urea was procured from USB (Cleveland, Ohio), and Ampicillin was purchased from Biochem Pharmaceutical Industries Limited (India). Guanidinium chloride (GdmCl) was from Serva (Heidelberg, Germany), Protease inhibitor cocktail was from ROCHE Diagnostics GmbH (Mannheim, Germany), Trizol was from Gibco BRL (Grand Island, NY), NaCl was from Qualigens Fine Chemicals (India). Mannitol was from Himedia laboratories (India). Ni-NTA matrix was obtained from QIAGEN (GmbH, Hilden). Other chemicals or reagents were from Sigma (St. Louis, MO). Water used in all reactions was obtained from MilliQ, Millipore (Bedford, MA).

### Cloning and expression of PrP (23-231) gene

We have cloned PrP (23-231) from mouse brain. Mouse brain was homogenized and total RNA was extracted using Trizol. Reverse transcription was carried out using MMLV RT and oligo dT. Using gene-specific primers (forward primer TGCCATATGAAA AAGCGGCCAAA-GCCTG and reverse primer GCTTTCGAATCAGCTGGATCTTCTCCCG), moPrP gene was amplified in MJ Research Peltier thermo cycler. Blunt end cloning was done using pMOS *Blue* blunt ended cloning kit (Amersham Pharmacia). Insert in pMOS *blue vector* and pET21a vector (Novagen) were digested with NdeI and HindIII and ligated by T4-DNA polymerase (Promega). *E. coli* DH5α strain was transfected with the plasmid construct. Positive colonies were screened with the help of ampicillin. The sequence of this construct was verified using 3700ABI automated DNA sequencer. Plasmid (PrP gene cloned in pET21a) from the positive colonies was isolated and transformed into *E.coli* Rosetta DE3 strain for protein expression.

### Purification of MoPrP (23-231)

MoPrP (23-231) was purified using a Ni-NTA column as described earlier [51] with minor modifications. Briefly, moPrP 23-231 (pET21a vector) was expressed in *E.coli* Rosetta DE3. After 12 h of induction, cells were harvested, lysed and centrifuged. The insoluble pellet was washed and dissolved in 8 M urea and 10 mM reduced glutathione and bound to Ni-NTA matrix. On-column oxidative folding of PrP was done by slow removal of the denaturant and the reducing agent. Protein was eluted in 1 M imidazole and was extensively dialyzed against MilliQ water and then concentrated. Aliquots of protein were stored at −70°C. The protein concentrations were estimated using the extinction coefficient of 2.70 at 280 nm for a 1 mg/ml solution of moPrP (23-231). This process yielded approx. 40 mg of protein from a 1 litre culture. The purity of the protein was checked using silver stained SDS-PAGE and western blot (using specific anti-prion antibody, Santacruz Biotechnology Inc., CA) and found it to be free of any contaminants.

### Photo-aggregation of prion protein

2.5 ml of 2.6 µM of recombinant mouse full-length prion protein was prepared in 50 mM of sodium phosphate buffer pH 7.4 and exposed to UV light in a 3 ml capacity 1 cm quartz cuvette with continuous stirring at room temperature (25°C). In all aggregation reactions, these parameters were kept invariant. Spectrofluorimeter Fluorolog-3 Model FL-3-22 with 450-W xenon light source and double grating excitation and emission monochromators from Horiba Jobin Yvon Inc. (Edison, NJ) was used to expose the protein to UV light and monitor its aggregation. Using the Datamax software provided by Jobin Yvon, we programmed the machine to expose the protein sample to wavelength of 290 nm light for one minute with 8 nm excitation bandpass and measure Rayleigh scattering within 15 seconds with excitation and emission monochromators at 465 nm with 5 nm bandpass. The measurements were carried out 30–40 times. We have exposed prion protein to light of various wavelengths such as 214, 350 and 400 nm and found no aggregation. For inhibition of aggregation, required amounts of antioxidants were added to the reaction prior to light exposure. Catalase enzyme (Sigma) is stored in 2.7 M ammonium sulphate. Mouse full-length prion protein aggregates in high ionic strength buffers. In order to avoid such high salt induced aggregation, we desalted the enzyme on a PD10 desalting column (Amersham). Activity of the desalted enzyme was determined using H_2_O_2_ assay. We have used 2.235 mU of catalase for further experiments.

### Amyloid formation of prion protein

Full-length mouse prion protein was converted to its amyloid form using procedure as described earlier [22] with minor modifications; Bocharova et al [22] formed fibrils from the unfolded protein. We, however, formed fibrils from the native state. In brief, to form amyloid fibrils *de novo*, recombinant PrP (43.4 µM and different dilutions as required) was incubated under amyloid forming condition, 1 M GdmHCl, 3 M urea, 150 mM NaCl in 20 mM phosphate buffer, pH 6.8, with continuous shaking at 600 rpm at 37°C. In the case of seeded reactions, seeds were made of fibrils of prion protein after sonication with 5 pulses of 2 second duration. Seed induced amyloid was formed by the addition of 5% (∼2.17 µM) of seeds (W/W) in amyloid forming conditions. Thioflavin T (ThT) fluorescence was monitored to follow the kinetics of fibril formation. Prion protein (0.434 µM) in 10 µM of ThT was used to measure kinetics of amyloid formation in HITACHI F4000 Fluorescence Spectrophotometer (with excitation and emission monochromator set at 445 nm and 485 nm respectively and a 10 nm band pass).

### Circular Dichroism studies

Far-UV CD spectra of different concentrations of unexposed and UV- exposed prion protein were recorded using a JASCO J-815 spectropolarimeter. Experiments were performed with different concentrations of prion protein in amyloid forming buffer (1 M GdmCl, 3 M Urea, and 150 mM NaCl in 20 mM phosphate buffer, pH 6.8) using a 0.01-cm path length cell. All spectra reported are the average of four accumulations and corrected for blank.

### Transmission Electron Microscopy (TEM)

A portion of the sample of prion protein fibrils was placed on Formavar/carbon-coated grids (300-mesh). Excess sample was removed and the grid was air-dried. The fibril-bearing grid was stained with 2% (w/v) uranyl acetate for 2 minutes. Images from randomly selected areas were captured on a film at 5,000–25,000× magnification on a JEOL (Tokyo, Japan) JEM-2100 LaB_6_ Transmission electron microscope with 100 kV accelerating voltage. Images were processed using Digital Micrograph version 3.11.0 from Gatan Inc. (Pleasanton, CA). Only background noise was removed from the acquired images. To calculate width of fibrils, the image was analyzed by Histogram analysis and full width at half maximum of the histogram peaks were taken as width of the fibrils.

### Atomic Force Microscopy

The samples were imaged with Digital Instruments Bioscope AFM with Nanoscope IV controller using silicon probe RTESP7 having a tip radius of 10 nm and a spring constant of 20–80 N/m (Veeco Metrology group, Santa Barbara, CA). Samples were deposited on mica adhered to ProbeON Plus Microscope slides (Fisher Scientific, USA) and washed repeatedly to prevent deposition of urea and guanidinium chloride. Imaging was done in dry tapping mode at a frequency of 285–300 kHz. All imaging experiments were performed at a scan rate of 0.8 Hz and a drive frequency of 285–300 kHz. Images (512×512) were processed in Nanoscope (R) III version 5.30r1. Each scan was subjected to second order flattening. Tilts were removed using planefit. Phase image was captured along with height image. Phase lag of the cantilever oscillation with that of the piezoelectric drive is used to generate phase images in tapping mode AFM [50]. The phase images depend on the composition, adhesion, friction, viscoelasticity [52], and hardness (stiffness) [53] of the sample. We have recoded phase images in tapping mode AFM with moderate tapping (Asp/A0 = ∼0.5). Phase image was obtained to find compactness of molecules under imaging. Compactness (or stiffness) refers to hardness or softness of the sample. Hard sample gives larger change in phase angle; soft samples in contrast lead to smaller changes in phase angle [53]. In order to calculate phase of the fibrils, the image was subjected to particle analysis, phase was obtained throughout the scan by calculating mean phase of arbitrary small squares of equal size spanning the area containing fibrillar mass. The process yields mean phase angle with standard error.
